# Quantification and Potential Viability of Human Noroviruses in Final Effluent from Wastewater Treatment Works in Pretoria, South Africa

**DOI:** 10.1007/s12560-024-09589-0

**Published:** 2024-03-31

**Authors:** V. V. Mabasa, W. B. van Zyl, M. B. Taylor, J. Mans

**Affiliations:** 1 Department of Medical Virology, Faculty of Health Sciences, University of Pretoria, Private Bag X323, Gezina, Pretoria, 0031 South Africa; 2 National Health Laboratory Service-Tshwane Academic Division, Pretoria, South Africa

**Keywords:** Norovirus, PMAxx™, RT-qPCR, Viability, South Africa

## Abstract

Growing global concerns over water scarcity, worsened by climate change, drive wastewater reclamation efforts. Inadequately treated wastewater presents significant public health risks. Previous studies in South Africa (SA) have reported high norovirus levels in final effluent and sewage-polluted surface water, indicating pathogen removal inefficiency. However, the viability of these virions was not explored. This study assessed human norovirus viability in final effluent from wastewater treatment works (WWTWs) in Pretoria, SA. Between June 2018 and August 2020, 200 samples were collected from two WWTWs, including raw sewage and final effluent. Norovirus concentrations were determined using in-house RNA standards. Viability of noroviruses in final effluent was assessed using viability RT-qPCR (vPCR) with PMAxx™-Triton X-100. There was no significant difference in GI concentrations between raw sewage (*p* = 0.5663) and final effluent (*p* = 0.4035) samples at WWTW1 and WWTW2. WWTW1 had significantly higher GII concentrations in raw sewage (*p* < 0.001) compared to WWTW2. No clear seasonal pattern was observed in norovirus concentrations. At WWTW1, 50% (7/14) of GI- and 64.9% (24/37) of GII-positive final effluent samples had no quantifiable RNA after vPCR. At WWTW2, the majority (92.6%, 25/27) of GII-positive final effluent samples showed a 100% RNA reduction post vPCR. PMAxx™-Triton X-100 vPCR provides a more accurate reflection of discharge of potentially viable noroviruses in the environment than standard RT-qPCR. Despite significant reductions in potentially viable noroviruses after wastewater treatment, the levels of potentially viable viruses in final effluent are still of concern due to the high initial load and low infectious dose of noroviruses.

## Introduction

The ongoing global climate change has a negative impact on the already limited potable water supply (Sivakumar, 2011). There are increasing efforts to reclaim and reuse wastewater (Randazzo et al., 2019). However, if not properly treated, wastewater poses a public health threat due to the presence of infectious human enteric viruses (Sano et al., 2016). Disposal of untreated and poorly treated wastewater into the environment often leads to contamination of clean water sources (Shrestha et al., 2018) and ultimately fresh produce (Cook et al., 2019) as well as filter-feeding bivalve molluscs (Razafimahefa et al., 2020). Consumption of food and water contaminated with faecal matter causes, among other diseases, acute gastroenteritis and human norovirus is one of the primary causative agents in these cases (Magana-Arachchi & Wanigatunge, 2020). Noroviruses are single-stranded, positive-sense RNA viruses classified within the genus *Norovirus* and family *Caliciviridae*. Human noroviruses are included in five genogroups (GI, GII, GIV, GVIII and GIX), which encompass 49 P-types and 35 genotypes based on their polymerase and complete capsid amino acid sequences, respectively (Chhabra et al., 2019). Viruses belonging to GI and GII are more common and cause the majority of infections and outbreaks (Vega et al., 2014). Noroviruses infect people of all ages globally (Lopman et al., 2016), however, children ≤ 5 years (Riera-Montes et al., 2018), the elderly (≥ 65 years) (Lindsay et al., 2015) and immunocompromised individuals (Green, 2014) are at high-risk of severe illness and developing complications. Both symptomatic and asymptomatic individuals shed high titres (> 10^10^ viral genome copies/gram; gc/g) of noroviruses in stools during the acute infection phase and can continue shedding in relatively low titres (< 10^3^ gc/g) long after the illness has resolved (Teunis et al., 2015). Consequently, noroviruses are abundant in wastewater and sewage surveillance has proven to be useful in identifying genotypes and strains circulating in communities in a given geographical area (Mabasa et al., 2022). Although noroviruses are hardy and can survive most treatment methods (Lopman et al., 2012; Ngazoa et al., 2008), a substantial amount of the virions get damaged by different food and water treatment procedures that use heat, high hydrostatic pressure, ultraviolet radiation and virucidal chemical agents (Ezzatpanah et al., 2022). Detection of viral RNA using the current gold standard test, real-time RT-qPCR, cannot differentiate intact and potentially infectious viruses from inactivated virions (Jeong et al., 2017). As noroviruses cannot be propagated in conventional monolayer cell cultures, a promising solution is viability RT-qPCR (vPCR), which employs pre-treatment of samples with propidium monoazide (PMA) or its derivative PMAxx™. This is an intercalating dye that penetrates damaged virions and covalently cross-links to nucleic acid through visible-light photoactivation, therefore, inhibiting their amplification (Razafimahefa et al., 2021). Addition of anionic detergents such as sodium deoxycholate (Canh et al., 2019) or Triton X-100 (Moreno et al., 2015) has been shown to enhance penetration of PMAxx™ into damaged virus capsids, thereby, increasing PMAxx’s efficacy. The use of vPCR can provide a more accurate hazard assessment of potential food and water contamination compared to RT-qPCR (Anfruns-Estrada et al., 2019; Razafimahefa et al., 2021). Norovirus contamination of fresh produce can be caused by infected food-handlers during final preparations (Derrick et al., 2021), but many outbreaks associated with produce have no identifiable contamination source (Hardstaff et al., 2018). It is possible that norovirus contamination during production may contribute to these untraceable outbreaks, and the virus can remain infectious on the produce for an extended period, which can lead to infections if consumed (Wu et al., 2023).  A previous study in the Gauteng and Free State provinces of South Africa (SA) have reported high concentrations of noroviruses in wastewater effluent and sewage-polluted surface water, which is suggestive of inefficient removal of potentially harmful pathogens (Mabasa et al., 2018). However, the integrity and infectivity of these norovirus virions was not investigated. In a more recent study in a different geographical region of South Africa (City of Tshwane, Gauteng) a wide diversity of noroviruses as well as novel norovirus recombinants were identified in raw sewage and final wastewater effluent from two wastewater treatment works (WWTWs) (Mabasa et al., 2022). As a follow-up investigation the viability of these noroviruses was assessed.

## Materials and Methods

### Virus Strains

Mengovirus MC0 (kindly provided by Prof Albert Bosch, University of Barcelona) was propagated and titrated in Vero cell culture. The viruses were harvested by three freeze–thaw cycles and purified using the Fast-Trap™ Virus Purification and Concentration Kit (Millipore, Billerica, MA, USA) according to the manufacturer’s instructions. Infectious viruses were enumerated by determining the 50% tissue culture infectious dose (TCID_50_) with eight wells per dilution and 200 μL of inoculum per well with the TCID_50_ calculator by Marco Blinder using the Spearman-Kärber method (Vieyres & Pietschmann, 2013). Purified mengovirus served as an extraction efficiency control for all experiments, following ISO 15216-1:2017 as a guideline (Lowther et al., 2019). Norovirus GI.4 and GII.4 positive stools were retrieved from – 20 °C storage and stool suspensions (10%, w/v) were prepared using phosphate-buffered saline (PBS; pH 7.2, [Sigma-Aldrich Co., St Louis, MO, USA]) and treated with 0.2 volumes of chloroform (Merck KGaA, Darmstadt, Germany) to remove PCR inhibitors. The aqueous phase was used for downstream applications.

### The Efficacy of PMAxx™ With and Without Triton X-100

The efficacy of PMAxx™ (Biotium Inc., Fremont, CA, USA) alone and in combination with 0.5% (v/v) Triton-X100 (Dow, Philadelphia, PA, USA) in penetrating damaged virions and preventing PCR amplification, was compared using a norovirus GII.4 stool suspension. The virus suspension was divided into two groups: (1) no-heat-treatment (Sub-groups A-C) and (2) heat-inactivated at 98 °C for 10 min (Sub-groups D-F). Sub-groups A and D were controls (no PMAxx™ added), sub-groups B and E were treated with 50 µM PMAxx™ + 0.5% (v/v) Triton-X100 and sub-groups C and F with 50 µM PMAxx™. Sub-group G was a PBS negative control. The PMAxx™-treated samples were mixed by vortexing for 10 s and incubated at room temperature (23–25 °C), protected from light for 15 min with brief vortexing at 5-min intervals. The samples were then transferred to a PMA-Lite™ LED photolysis device (Biotium, Fremont, CA, USA) and incubated for 30 min at room temperature, protected from external light to photoactivate the dye. Thereafter total nucleic acids were extracted from 1 mL of each sample using the QIAamp UltraSens Virus Kit (Qiagen, Valencia, CA, USA), according to the manufacturer’s instructions. Nucleic acid was eluted in 100 µL nuclease-free water and stored at − 80 °C. Within 24 h, norovirus GII vPCR was performed in triplicate using a QuantiFast Pathogen RT-PCR + IC Kit (Qiagen) with published probes and primers (Table 1). The average cycle threshold (Ct) values were compared to assess the ability of PMAxx™ and Triton X-100 to inhibit nucleic acid amplification and to improve the permeability of damaged virions, respectively.

**Table 1:** Conventional PCR and real-time RT-PCR primers, probes and amplification conditions

Target		Primer	Sequence (5ʹ–3ʹ)*	Polarity	Location	References	Amplification conditions
Conventional PCR	Norovirus GI	T7GI.4F	**TAATACGACTCACTATAGGG**TTCCAGGGGAGGC	+	5172–5184^a^	This study	** × 40 cycles** 98 °C, 10 s 50 °C, 30 s 72 °C, 40 s
		GI.4R2	CCAGTCCAACCCAGCCATTG	−	5657–5677^a^	This study	72 °C, 5 min
	Norovirus GII	T7GII.4F	**TAATACGACTCACTATAGGG**CAAGAGTCAATGTTTAGGTGGATGAG	+	5003–5028*	(Moore & Jaykus, 2017)	** × 40 cycles** 98 °C, 10 s 55 °C, 30 s 72 °C, 40 s
		GII.4R2	GTTGGGAAATTCGGTGGGACTG	−	5452–5473*	(Moore & Jaykus, 2017)	72 °C, 5 min
Real-time RT-qPCR	Norovirus GI	QNIF4	CGCTGGATGCGNTTCCAT	+	5291–5308^a^	(da Silva et al., 2007)	50 °C, 20 min 95 °C, 5 min
		NV1LCR	CCTTAGACGCCATCATCATTTAC	−	5354–5376^a^	(Svraka et al., 2007)	** × 45 cycles** 95 °C, 15 s 55 °C, 30 s 65 °C, 30 s
		NVGG1	FAM-TGGACAGGAGAYCGCRATCT-TAMRA	+	5321–5340^a^	(Svraka et al., 2007)	
	Norovirus GII	QNIF2	ATGTTCAGRTGGATGAGRTTCTCWGA	+	5012–5037*	(Loisy et al., 2005)	50 °C, 20 min 95 °C, 5 min
		COG2R	TCGACGCCATCTTCATTCACA	−	5080–5100*	(Kageyama et al., 2003)	** × 45 cycles** 95 °C, 15 s 60 °C, 30 s 65 °C, 30 s
		QNIFS	FAM-AGCACGTGGGAGGGCGATCG-TAMRA	+	5042–5061*	(Loisy et al., 2005)	
	Mengovirus	Mengo110F	GCGGGTCCTGCCGAAAGT	+	110–127^b^	(Pintó et al., 2009)	50 °C, 20 min 95 °C, 5 min
		Mengo209R	GAAGTAACATATAGACAGACGCACAC	−	245–270^b^	(Pintó et al., 2009)	** × 45 cycles** 95 °C, 15 s 60 °C, 30 s 65 °C, 30 s
		Mengo147	MGB-ATCACATTACTGGCCGAAGC-TAMRA	+	208–227^b^	(Pintó et al., 2009)	

*Degeneracy code: Y = C/T; R = A/G; W = A/T; I = Inosine; N = any base. Bold bases = T7 promoter sequence. Probe labels: 6-carboxy fluorescein (FAM), minor groove binder (MGB) and 6-carboxy-tetramethylrhodamine (*TAMRA*). Location based on corresponding nucleotide position of M87661 (^a^), X86557 (*) and L22089 (^b^)

### Conventional RT-PCR and In-Vitro Transcription for RNA Standards

Viral RNA was extracted from the norovirus GI.4 and GII.4 stool suspensions using a QIAamp Viral RNA Kit (Qiagen) according to manufacturer’s instructions. Complementary DNA (cDNA) was synthesised from 10 µL of RNA using 30 µM random hexamers (Thermo Scientific, Waltham, MA, USA) and ProtoScript® II reverse transcriptase (New England Biolabs Inc., Ipswich, MA, USA) as specified by the manufacturer. Subsequently, 5 µL of cDNA was used as template for a one-round of PCR amplification using EmeraldAmp® Max HS PCR Master Mix (Takara, Shiga, Japan) to introduce a T7 promoter sequence (Metabion, Planegg, Germany) (Table 1). The PCR products were purified using Agencourt AMPure XP beads (Beckman Coulter, Brea, CA, USA) according to manufacturer’s instructions. The amplicon concentration was adjusted to between 2 µg/µL and 3 µg/µL. Four in vitro transcription reactions were prepared using MEGAshortscript™ T7 Transcription Kit (Invitrogen, Vilnius, Lithuania) according to manufacturer’s instructions. Briefly, a 20 μL reaction comprised of 2 μL T7 Reaction buffer, 8 μL T7 RNA nucleotide mix, 2 μL T7 Enzyme mix, 6 μL nuclease-free water and 2 μL prepared DNA template and the reactions were incubated at 37 °C for four hours. The amplification conditions for all PCR assays are described in Table 1.

### RNA Purification

Post *in-vitro* transcription, template DNA was degraded using Turbo™ DNase. Briefly, 5 µL 1X Turbo™ DNase (Invitrogen) buffer, 2 µL Turbo™ DNase (2 U/µL) (Invitrogen) and nuclease-free water were mixed to a final volume of 50 µL and incubated at 37 °C for 30 min. To maximise DNA degradation, the procedure was repeated. The RNA transcripts were further purified using the Direct-zol™ RNA Miniprep Plus Kit (Zymo Research, Irvine, CA, USA) according to manufacturer’s instructions. This kit includes an additional on-column DNase digestion. The purified RNA was eluted using nuclease-free water in a final volume of 50 µL. All centrifugations were performed at 16 000 × g.

### Assessing RNA Purity and Concentration

The transcripts (5 µL RNA) were subjected to a one-step RT-qPCR using a QuantiFast Pathogen RT-PCR + IC Kit with published norovirus GI and GII probes and primers (Table 1). The assay was performed without the RT enzyme to assess the amount of DNA in the in vitro transcription products. The kit’s internal control was used to monitor the efficiency of target amplification and all real-time RT-qPCR assays in the study were performed on a QuantStudio™ 5 real-time system (Applied Biosystems, Foster City, CA, USA). The RNA concentration was measured using the Qubit™ RNA XR Assay Kit (Invitrogen, Eugene, OR, USA) on Qubit® 3.0 Fluorometer and the RNA stocks were aliquoted and stored at – 80 °C.

### Norovirus and Mengovirus Quantification by RT-qPCR in Wastewater Samples

From June 2018 to August 2020, bi-weekly wastewater samples, including 1 L of raw sewage and 10 L of final effluent, were collected from two WWTWs in Pretoria, within the City of Tshwane Metropolitan Municipality, South Africa. The sampling took place consistently between 7 and 9 am, capturing the peak morning flow. The WWTW1 and WWTW2 differ primarily in their treatment processes and influent control. The WWTW1 exclusively employs an activated sludge process but is not able to regulate the incoming flow, leading to its current operation at 103% of its hydraulic capacity. In contrast, WWTW2 treats a portion of the incoming flow with biological trickling filters (16% of flow) alongside an activated sludge process (84% of flow) and is able to control the incoming flow to below its design capacity at 68% capacity utilization (DWS, 2023a). Both facilities utilise chlorine as the final treatment step before discharging treated effluent into rivers. Viral concentrates prepared in the Mabasa et al., 2022 study were used in this investigation. Skimmed milk flocculation was used for recovery of raw sewage samples and glass-wool adsorption elution for final effluent samples. Viral concentrations in norovirus-positive raw sewage samples (89/100) and final effluent samples (73/100) (Mabasa et al., 2022) were determined based on norovirus GI and GII standard curves generated with RNA transcripts. The mengovirus standard curve was generated using RNA extracted from viral stock propagated in cell culture. Ten-fold serial dilutions of RNA were prepared using nuclease-free water in triplicate for each virus and RT-qPCR assays were performed using a QuantiFast Pathogen RT-PCR + IC Kit with published primers and probes (Table 1), according to manufacturer’s instructions.

### Norovirus Viability PCR in Wastewater Samples

Sixty-six of 73 norovirus-positive final effluent samples (2 GI, 49 GII, and 15 GI + GII) had sufficient material (minimum 1 mL viral concentrate) for viability experiments. These 1 mL viral concentrates were thawed and treated with 0.2 volumes of chloroform as previously described (Mabasa et al., 2022). Triton X-100 was then added to the samples at a final concentration of 0.5%, followed by incubation at room temperature for 10 min with brief vortexing at 2 min intervals. PMAxx™ was subsequently added at a final concentration of 50 μM and vigorously vortexed for 10 s before incubation in the dark at room temperature for 15 min, during which the samples were briefly vortexed at 5 min intervals. The samples were then transferred to a PMA-Lite™ LED photolysis device protected from external light and incubated for 30 min. Total nucleic acids were extracted from the samples as previously described (Mabasa et al., 2022). As a control, ten viral concentrates from norovirus-positive raw sewage samples were randomly selected, treated with Triton X-100 and PMAxx™, and subjected to nucleic acid extraction and vPCR. The percentage difference in norovirus concentration following PMAxx™ treatment was calculated by dividing the difference between concentrations with and without PMAxx™ treatment by the initial norovirus concentration without PMAxx™ treatment. The resulting quotient was then multiplied by 100.

### Statistical Analysis

Results were statistically analysed and significance of differences between norovirus GI and GII concentrations at both sampling sites was determined with a two-sided t-test. In all cases, a value of *p* < 0.05 was considered to be significant (https://openepi.com/Mean/t_testMean.htm).

## Results

### PMAxx™ Efficacy With and Without Triton X-100

The effect of Triton X-100 on PMAxx*™* efficacy was determined using a norovirus GII.4 stool suspension and the assay successfully detected norovirus GII in both the no-heat-treatment and the heat-inactivated groups (Table 2). There was a significant 1.5 log difference in Ct values between sub-groups A and B (*p* < 0.05). Sub-groups A and C also had a significant difference in Ct values (*p* < 0.05), however, no significant difference in Ct values was observed between sub-groups B and C (*p* = 0.18). There was a significant difference (*p* < 0.05) equivalent to 2.7 logs in Ct values between sub-groups D and E. Sub-groups D and F had a significant 1.8 log difference (*p* < 0.05), while sub-groups E and F had no significant difference (*p* = 0.07). There was no significant difference in average Ct values between sub-groups A and D (*p* = 0.19), and between sub-groups C and F (*p* = 0.10). Sub-groups B and E had a significant 0.9 log difference (*p* < 0.05). The control group, sub-group G had no detectable norovirus RNA as expected. Due to apparent added benefits, wastewater samples were subjected to pre-treatment with 0.5% Triton X-100 before undergoing PMAxx™ treatment.

**Table 2:** The effect of Triton X-100 on PMAxx*™* viability PCR efficacy determined using a heat-inactivated norovirus GII.4 stool suspension

Group	Treatment	Sub-group	Treatment replicates	RT-qPCR replicates	Ct	Average Ct
No-heat-treatment	no PMAxx™ added	A	1	1	26.27	27 ± 1.47
				2	27.81	
				3	27.41	
			2	4	28.74	
				5	25.33	
				6	27.90	
			3	7	26.25	
				8	27.34	
				9	24.00	
	0.5% Triton X-100 + 50 µM PMAxx™	B	4	10	33.65	33 ± 1.55
				11	33.67	
				12	33.77	
			5	13	34.74	
				14	34.86	
				15	33.69	
			6	16	31.24	
				17	30.85	
				18	31.20	
	50 µM PMAxx™	C	7	19	31.98	32 ± 0.84
				20	34.19	
				21	33.39	
			8	22	32.08	
				23	31.76	
				24	32.83	
			9	25	31.76	
				26	31.92	
				27	32.50	
Heat-inactivated at 98 °C for 10 min	no PMAxx™ added	D	10	28	26.05	27 ± 0.84
				29	25.72	
				30	25.68	
			11	31	26.19	
				32	26.65	
				33	26.02	
			12	34	27.58	
				35	27.44	
				36	27.86	
	0.5% Triton X-100 + 50 µM PMAxx	E	13	37	34.45	36 ± 1.23
				38	35.84	
				39	36.47	
			14	40	34.29	
				41	35.94	
				42	37.93	
			15	43	34.12	
				44	36.12	
				45	35.02	
	50 µM PMAxx™	F	16	46	29.43	33 ± 2.85
				47	30.11	
				48	31.88	
			17	49	32.85	
				50	31.82	
				51	31.81	
			18	52	35.54	
				53	35.90	
				54	37.98	

### RNA Standard Curves

Post RNA purification, template DNA could not be detected (Ct ≥ 40) by qPCR. The measured norovirus GI, GII and mengovirus RNA concentrations were 1.6 × 10^7^ gc/µL, 1.9 × 10^7^ gc/µL and 1.84 × 10^10^ gc/µL, respectively. All RNA standard curves showed satisfactory negative linearity with a wide dynamic range. The norovirus GI standard curve had a slope of -3.34, the y-intercept was 43.26, and the coefficient of determination (*R*^*2*^) was 0.98 and an efficiency of 98.18%. The norovirus GII standard curve had a slope of − 3.32, the y-intercept was 42.76, the *R*^*2*^ value of 0.99 and an efficiency of 99%. The mengovirus standard curve had a slope of − 3.42, the y-intercept was 47.12, the *R*^*2*^ value of 0.96 and an efficiency of 95.91%.

### Norovirus Quantification by RT-qPCR in Wastewater Samples

Noroviruses were detected and quantified in 89% (89/100) of raw sewage and 73% (73/100) final effluent samples. The calculated mengovirus-based extraction efficiencies varied between samples, however, no samples were re-extracted as they all complied with the guidelines indicated in the ISO 15216-1:2017 (Lowther et al., 2019). The virus concentrations were not adjusted according to calculated extraction efficiencies considering that back-calculation is not recommended (Haramoto et al., 2018).

**Fig. 1:**
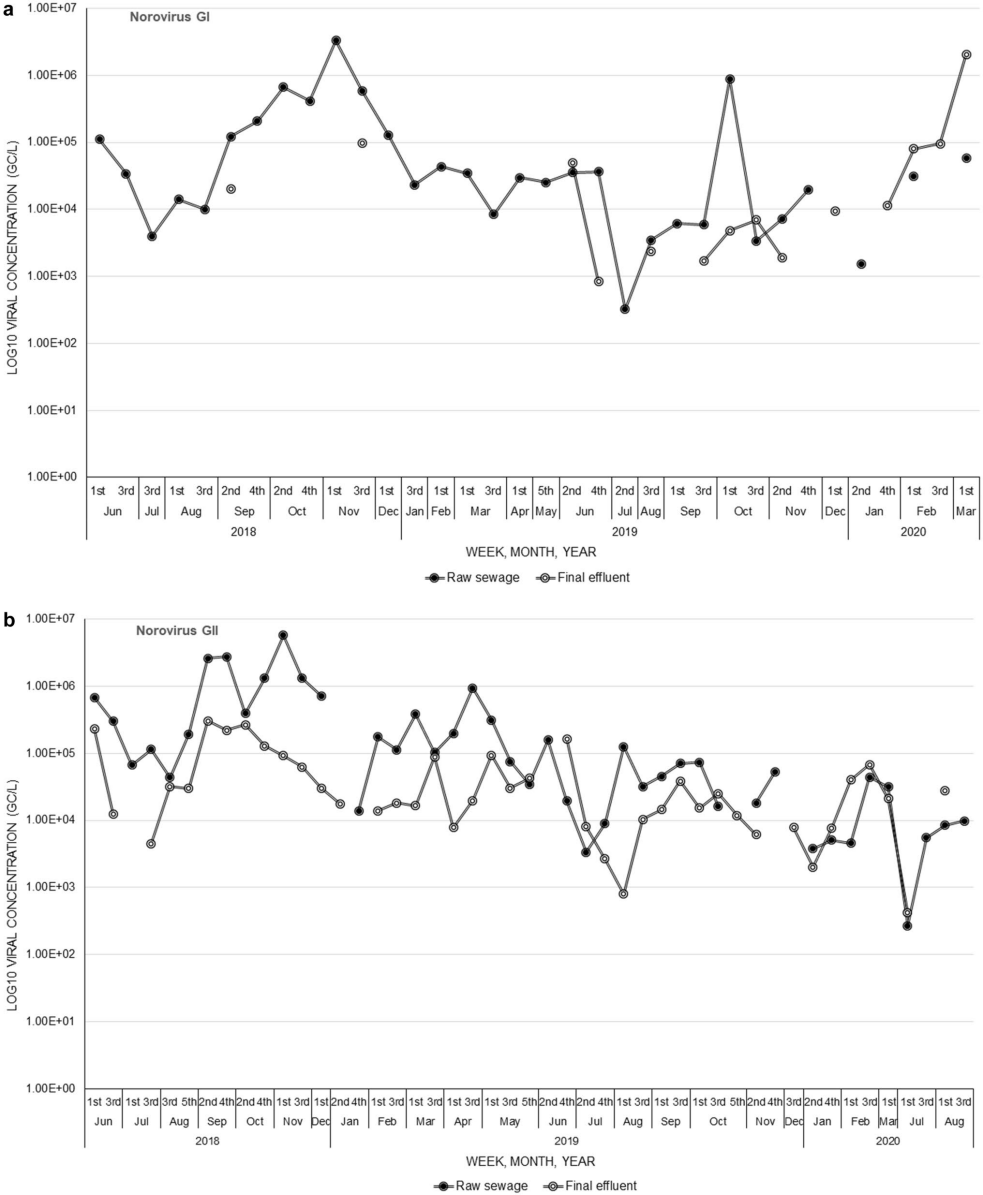
Norovirus GI (**a**) and GII (**b**) Log_10_ concentrations in genome copies per litre (gc/L) in raw sewage and final effluent samples for wastewater treatment works 1 (WWTW1)

The median extraction efficiency was 20% and the interquartile range was 86%. Of the norovirus-positive raw sewage samples 71.9% (64/89) were GI and 95.5% (85/89) were GII, while in the final effluent samples, 21.9% (16/73) were GI and 95.9% (70/73) GII. At WWTW1, the norovirus GI concentrations in raw sewage ranged between 3.22 × 10^2^ gc/L and 6.68 × 10^5^ gc/L with an average of 1.18 × 10^5^ ± 2.21 × 10^5^ gc/L, and from 8.30 × 10^2^ gc/L to 9.66 × 10^4^ gc/L with an average of 2.93 × 10^4^ ± 3.74 × 10^4^ gc/L in final effluent samples (Fig. 1a). The GII concentrations in raw sewage ranged between 2.71 × 10^2^ gc/L and 5.74 × 10^6^ gc/L with an average of 4.39 × 10^5^ ± 1.02 × 10^6^ gc/L, and in final effluent concentrations ranged between 4.19 × 10^2^ gc/L and 3.04 × 10^5^ gc/L with an average of 5.45 × 10^4^ ± 7.65 × 10^4^ gc/L (Fig. 1b). There was a significant difference (*p* < 0.001) in average concentration of both GI and GII viruses between the raw sewage and final effluent samples, with the latter having a lower concentration. Interestingly, the average concentration of GII was significantly higher (*p* = 0.01) than that of GI in final effluent samples.

**Fig. 2:**
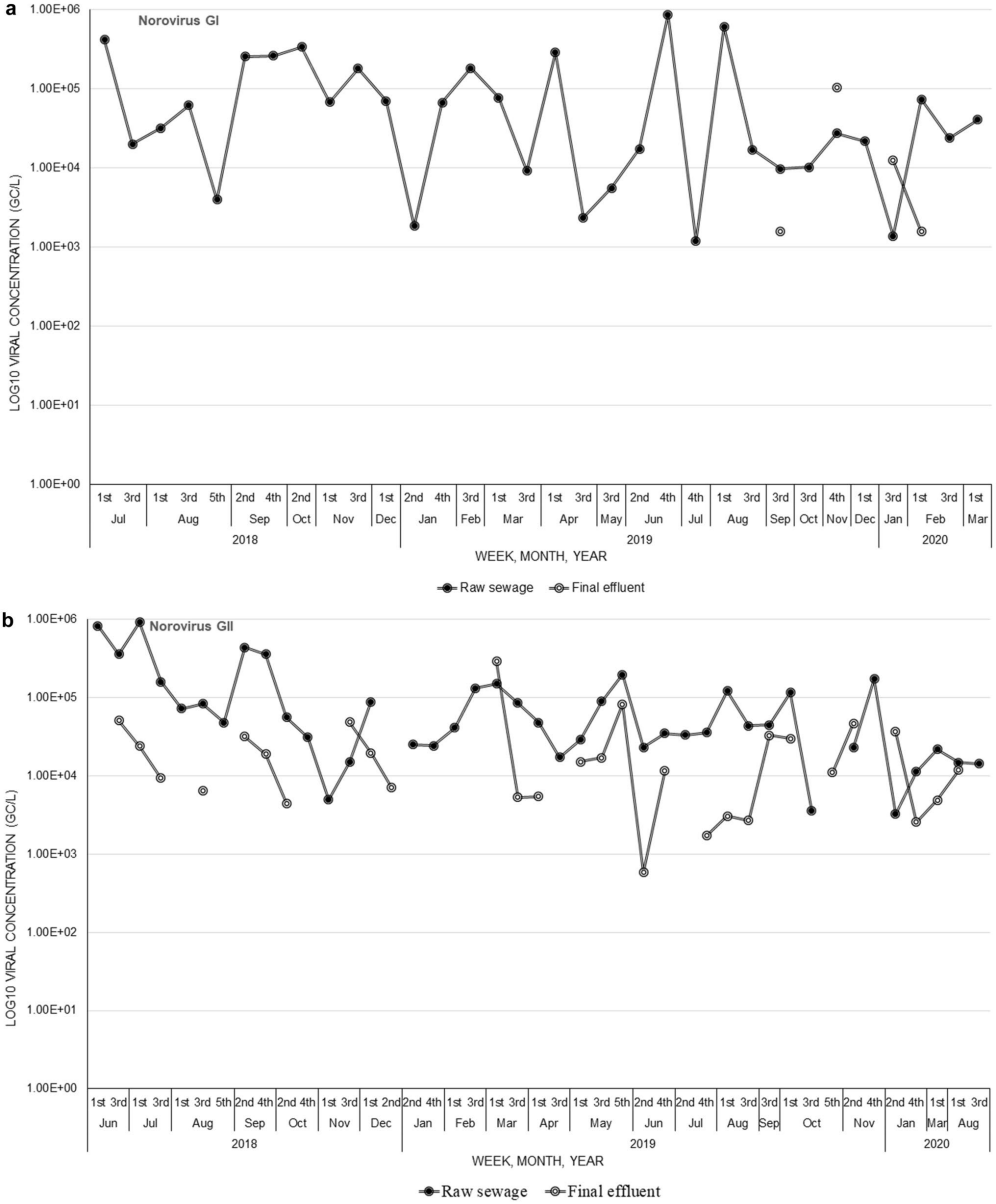
Norovirus GI (**a**) and GII (**b**) Log_10_ concentrations in genome copies per litre (gc/L) in raw sewage and final effluent samples for wastewater treatment works 2 (WWTW2)

At WWTW2, norovirus GI concentrations in raw sewage samples ranged between 1.38 × 10^3^ gc/L and 8.72 × 10^5^ gc/L with an average of 1.27 × 10^5^ ± 1.99 × 10^5^ gc/L, while in final effluent samples was between 1.57 × 10^3^ gc/L and 1.05 × 10^5^ gc/L with an average of 3.02 × 10^4^ ± 5.01 × 10^4^ gc/L (Fig. 2a). The GII concentrations in raw sewage were between 3.23 × 10^3^ gc/L and 9.22 × 10^5^ gc/L with an average of 1.23 × 10^5^ ± 2.00 × 10^5^ gc/L and ranged from 5.86 × 10^2^ gc/L to 2.91 × 10^5^ gc/L with an average of 2.87 × 10^4^ ± 5.39 × 10^4^ gc/L in final effluent samples (Fig. 2b). Norovirus concentrations were significantly lower in final effluent compared to raw sewage samples for both GI (*p* = 0.04) and GII (*p* < 0.001). Overall, there was no significant difference in the average norovirus GI concentrations in raw sewage (*p* = 0.5663) and final effluent (*p* = 0.4035) samples between WWTW1 and WWTW2. However, WWTW1 had significantly (*p* < 0.001) higher concentrations of norovirus GII in raw sewage compared to WWTW2. Although WWTW1 had a higher average norovirus GII concentration in final effluent compared to WWTW2, the difference was not significant (*p* = 0.0553). There was no clear seasonality pattern in the concentrations recorded for both GI and GII at both WWTWs.

### Norovirus vPCR

The samples had varying extraction efficiencies, however, all were above 1%, which is acceptable according to the ISO 15216–1:2017 (Lowther et al., 2019). The median extraction efficiency for PMAxx™-Triton X-100 treated samples was 17.5% and the interquartile range was 76%. The majority (59%; 39/66) of final effluent samples with detectable and quantifiable norovirus were from WWTW1 and they comprised of 2 GI, 25 GII and 12 with both GI and GII. The remaining 41% (27/66) were samples from WWTW2 and they comprised of 26 GII and 1 with both GI and GII. The ten raw sewage samples included as control comprised of 1 GI, 1 GII and 2 with both GI and GII from WWTW1, and 3 GII and 3 with both GI and GII from WWTW2. After the PMAxx™-Triton X-100 treatment, the norovirus GI and GII concentrations in raw sewage showed a reduction of between 92.1% and 100%, except in one sample where the reduction in GI concentration was only 56%. The norovirus concentrations ranged between 3.79 × 10^2^ gc/L and 5.10 × 10^3^ gc/L at WWTW1 (Table 3) and from 3.09 × 10^2^ gc/L to 1.83 × 10^5^ gc/L at WWTW2 (Table 4).

**Table 3:** Norovirus GI and GII concentrations measured in genome copies per litre in raw sewage and final effluent samples from WWTW1 with and without PMAxx + Triton X-100

Final Effluent								
Year	Month	Week	Norovirus GII			Norovirus GI		
			No treatment [gc/L]	PMAxx + Triton X-100 [gc/L]	Difference %	No treatment [gc/L]	PMAxx + Triton X-100 [gc/L]	Difference %
2018	Jun	1st	2.32E + 05	0	↓100.0%	–	–	–
		3rd	1.25E + 04	0	↓100.0%	–	–	–
	Jul	1st	–	0	–	–	–	–
		3rd	4.51E + 03	0	↓100.0%	–	–	–
	Aug	3rd	3.20E + 04	0	↓100.0%	–	–	–
		5th	2.99E + 04	0	↓100.0%	–	–	–
	Sep	2nd	3.04E + 05	0	↓100.0%	–	–	–
		4th	2.19E + 05	0	↓100.0%	–	–	–
	Oct	2nd	2.67E + 05	3.33E + 02	↓99.9%	9.66E + 04	1.87E + 03	↓98.1%
		4th	1.30E + 05	0	↓100.0%	–	–	–
	Nov	1st	9.16E + 04	0	↓100.0%	–	–	–
		3rd	6.21E + 04	7.91E + 02	↓98.7%	9.66E + 04	0	↓100.0%
	Dec	1st	3.02E + 04	0	↓100.0%	–	–	–
		2nd	–	–	–	–	–	–
2019	Jan	2nd	1.76E + 04	0	↓100.0%	–	–	–
		4th	–	–	–	–	–	–
	Feb	1st	1.38E + 04	2.36E + 03	↓83.0%	–	–	–
		3rd	1.83E + 04	0	↓100.0%	–	–	–
	Mar	1st	1.03E + 05	2.07E + 02	↓99.8%	8.43E + 03	0	↓100.0%
		3rd	8.58E + 04	0	↓100.0%	9.23E + 03	0	↓100.0%
	Apr	1st	9.43E + 05	0	↓100.0%	–	–	–
		3rd	1.73E + 04	0	↓100.0%	2.35E + 03	0	↓100.0%
	May	1st	7.52E + 04	0	↓100.0%	–	–	–
		5th	1.96E + 05	0	↓100.0%	–	–	–
	Jun	2nd	–	–	–	4.91E + 04	1.09E + 04	↓77.8%
		4th	1.64E + 05	0	↓100.0%	8.30E + 02	0	↓100.0%
	Jul	2nd	8.05E + 03	4.53E + 03	↓43.8%	–	–	–
		4th	2.67E + 03	0	↓100.0%	–	–	–
	Aug	1st	8.03E + 02	2.03E + 03	**↑152.4%**	–	–	–
		3rd	1.03E + 04	9.09E + 03	↓12.0%	2.38E + 03	2.41E + 03	**↑1.3%**
	Sep	1st	1.44E + 04	9.46E + 05	**↑6451.6%**	–	–	–
		3rd	3.85E + 04	2.63E + 04	↓31.7%	–	–	–
	Oct	1st	1.54E + 04	4.11E + 03	↓73.3%	4.82E + 03	8.70E + 03	**↑80.4%**
		3rd	2.52E + 04	0	↓100.0%	6.95E + 03	0	↓100.0%
		5th	1.18E + 04	0	↓100.0%	–	–	–
	Nov	2nd	6.15E + 03	0	↓100.0%	1.88E + 03	0	↓100.0%
		4th	–	–	–	–	–	–
	Dec	1st	–	–	–	9.43E + 03	1.01E + 04	**↑6.6%**
		3rd	7.77E + 03	3.55E + 03	↓54.4%	–	–	–
2020	Jan	2nd	2.02E + 03	0	↓100.0%	–	–	–
		4th	7.68E + 03	0	↓100.0%	–	–	–
	Feb	1st	4.01E + 04	7.00E + 04	**↑74.4%**	8.00E + 04	1.19E + 06	**↑1386.1%**
		3rd	6.74E + 04	6.80E + 04	**↑0.9%**	9.55E + 04	1.01E + 05	**↑5.7%**
Positive after PMAxx + Triton X-100			35%			50%		

Raw sewage								
Year	Month	Week	Norovirus GII			Norovirus GI		
			No treatment [gc/L]	PMAxx + Triton X-100 [gc/L]	Difference %	No treatment [gc/L]	PMAxx + Triton X-100 [gc/L]	Difference %
2018	Jun	3rd	3.02E + 05	0	↓100.0%	3.40E + 04	3.79E + 02	↓98.9%
	Jul	1st	6.70E + 04	5.10E + 03	↓92.4%	–	–	–
	Dec	1st	–	–	–	1.27E + 05	4.02E + 03	↓96.8%
2019	Mar	1st	3.81E + 05	4.92E + 03	↓98.7%	3.46E + 04	0	↓100.0%
Positive after PMAxx + Triton X-100			**67%**			**67%**		

– Not applicable

**Table 4:** Norovirus GI and GII concentrations measured in genome copies per litre in raw sewage and final effluent samples from WWTW2 with and without PMAxx + Triton X-100

Final Effluent								
Year	Month	Week	Norovirus GII			Norovirus GI		
			No treatment [gc/L]	PMAxx + Triton X-100 [gc/L]	Difference %	No treatment [gc/L]	PMAxx + Triton X-100 [gc/L]	Difference %
2018	Jun	3rd	5.07E + 04	0	↓100.0%	–	–	–
	Jul	1st	2.41E + 04	0	↓100.0%	–	–	–
		3rd	9.38E + 03	0	↓100.0%	–	–	–
	Aug	3rd	6.42E + 03	0	↓100.0%	–	–	–
	Sep	2nd	3.18E + 04	0	↓100.0%	–	–	–
		4th	1.90E + 04	0	↓100.0%	–	–	–
	Oct	2nd	4.48E + 03	0	↓100.0%	–	–	–
	Nov	3rd	4.88E + 04	0	↓100.0%	–	–	–
	Dec	1st	1.96E + 04	0	↓100.0%	–	–	–
		2nd	7.05E + 03	0	↓100.0%	–	–	–
2019	Mar	1st	8.83E + 04	0	↓100.0%	–	–	–
		3rd	5.34E + 03	0	↓100.0%	–	–	–
	Apr	1st	1.96E + 04	0	↓100.0%	–	–	–
		3rd	1.03E + 04	1.28E + 03	↓87.6%	–	–	–
	May	1st	3.05E + 04	0	↓100.0%	–	–	–
		5th	8.23E + 04	0	↓100.0%	–	–	–
	Jun	2nd	5.86E + 02	0	↓100.0%	–	–	–
		4th	1.15E + 04	0	↓100.0%	–	–	–
	Jul	4th	1.73E + 03	0	↓100.0%	–	–	–
	Aug	1st	3.05E + 03	0	↓100.0%	–	–	–
		3rd	2.70E + 03	0	↓100.0%	–	–	–
	Sep	3rd	3.31E + 04	0	↓100.0%	–	–	–
	Oct	1st	3.01E + 04	0	↓100.0%	–	–	–
		5th	1.10E + 04	0	↓100.0%	–	–	–
	Nov	4th	4.68E + 04	0	↓100.0%	1.05E + 05	0	↓100.0%
2020	Jan	2nd	3.65E + 04	8.37E + 03	↓77.1%	–	–	–
		4th	2.57E + 03	0	↓100.0%	–	–	–
Positive after PMAxx + Triton X-100			7%			0%		

Raw sewage								
Year	Month	Week	Norovirus GII			Norovirus GI		
			No treatment [gc/L]	PMAxx + Triton X-100 [gc/L]	Difference %	No treatment [gc/L]	PMAxx + Triton X-100 [gc/L]	Difference %
2018	Jun	1st	8.30E + 05	4.51E + 03	↓99.5%	–	–	–
		3rd	5.07E + 04	1.33E + 03	↓97.4%	–	–	–
	Jul	1st	9.22E + 05	2.24E + 03	↓99.8%	4.20E + 05	1.83E + 05	↓56.4%
2019	Feb	3rd	1.32E + 05	1.04E + 04	↓92.1%	1.84E + 05	1.31E + 04	↓92.9%
	May	5th	1.96E + 05	9.90E + 03	↓95.0%	–	–	–
	Jul	4th	3.57E + 04	3.09E + 02	↓99.1%	1.18E + 03	0	↓100.0%
Positive after PMAxx + Triton X-100			100%			67%		

– Not applicable

**Fig. 3:**
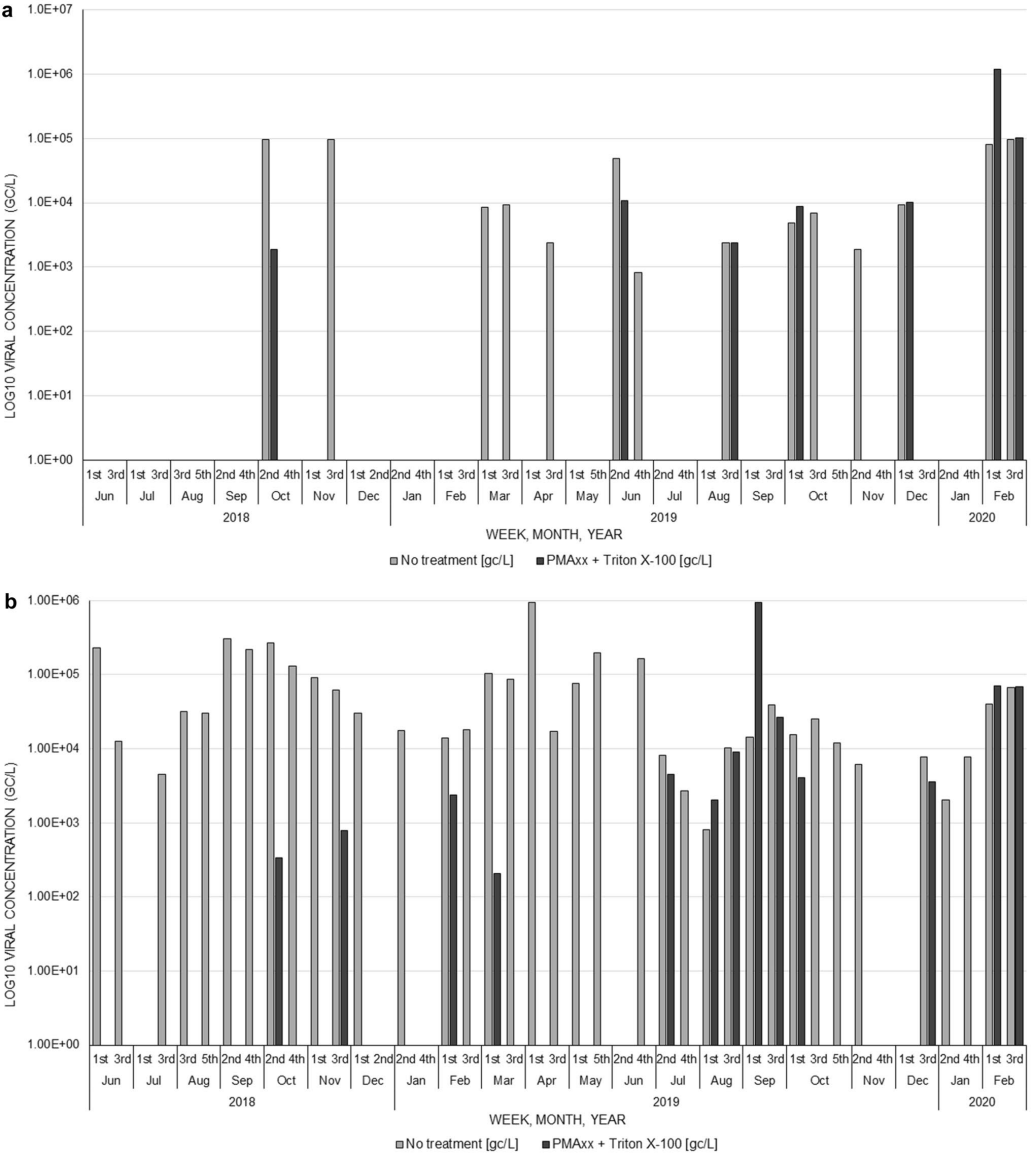
Norovirus GI (**a**) and GII (**b**) log_10_ concentrations in genome copies per litre (gc/L) before and after PMAxx™-Triton X-100 treatment in final effluent samples for wastewater treatment works 1 (WWTW1)

From WWTW1, 50% (7/14) of the GI positive final effluent samples had no quantifiable RNA after PMAxx™-Triton X-100 treatment (Table 3). Two samples had between 1.83 × 10^3^ gc/L and 1.09 × 10^4^ gc/L as a result of an average reduction of 6.65 × 10^4^ ± 3.99 × 10^4^ gc/L (Fig. 3a). Five samples had an average increase of 2.24 × 10^5^ ± 4.95 × 10^5^ gc/L with concentrations between 2.41 × 10^3^ gc/L and 1.19 × 10^6^ gc/L. The majority (64.9%, 24/37) of the GII positive samples had no quantifiable RNA after PMAxx™-Triton X-100 treatment (Fig. 3b). Nine of the samples had an average reduction of 5.27 × 10^4^ ± 8.72 × 10^4^ gc/L and their concentrations ranged between 2.07 × 10^2^ gc/L and 2.63 × 10^4^ gc/L. The remaining four samples had an average increase of 2.41 × 10^5^ ± 4.60 × 10^5^ gc/L with concentrations ranging from 5.85 × 10^2^ gc/L to 9.31 × 10^5^ gc/L. Overall, at WWTW1, there was no significant difference in the average reduction (*p* = 0.715) and measured concentrations (*p* = 0.109) between norovirus GI and GII after the PMAxx™-Triton X-100 treatment.

**Fig. 4:**
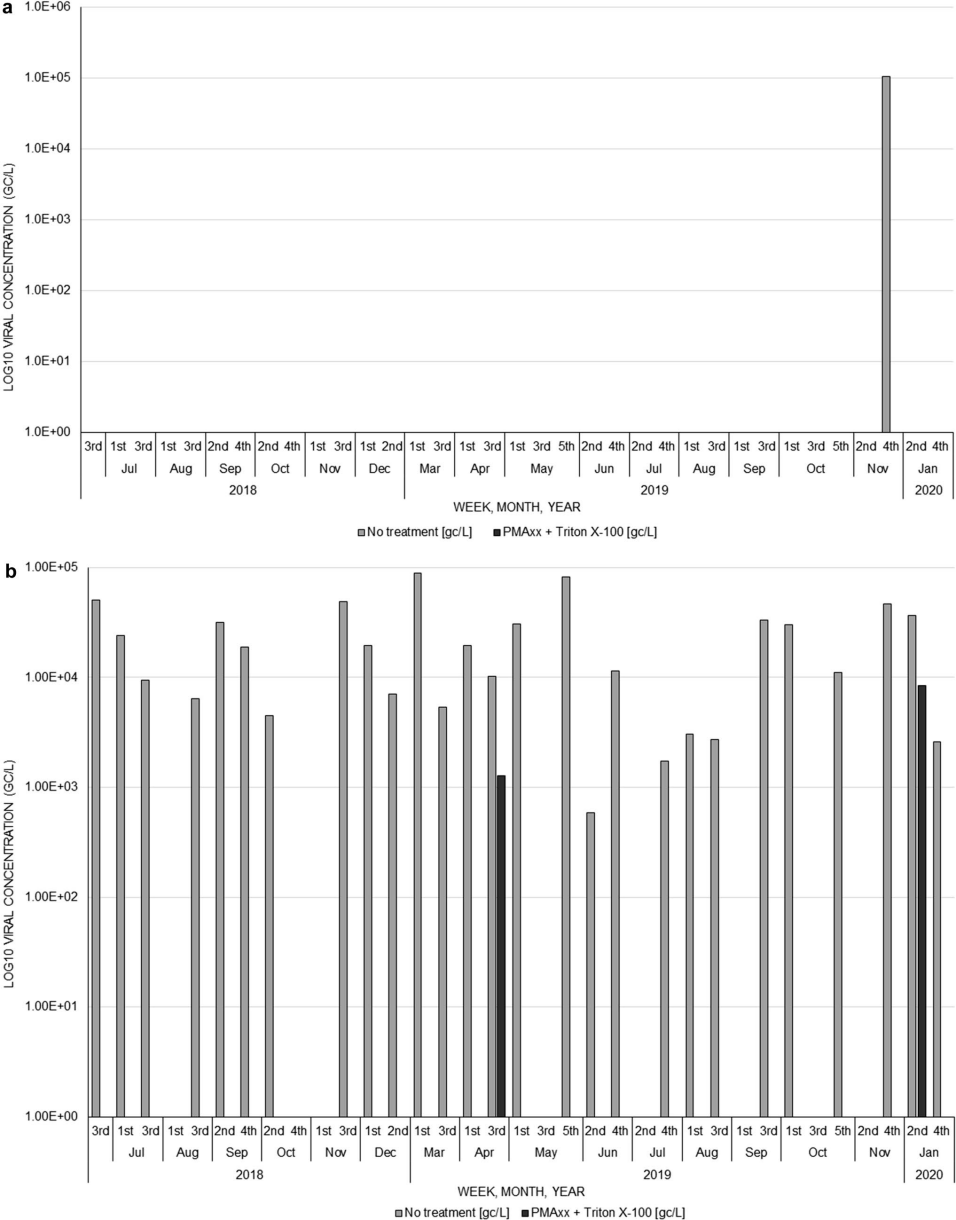
Norovirus GI (**a**) and GII (**b**) log_10_ concentrations in genome copies per litre (gc/L) before and after PMAxx™-Triton X-100 treatment in final effluent samples for wastewater treatment works 2 (WWTW2)

There was only one norovirus GI positive final effluent sample from WWTW2 and no RNA could be detected and quantified after the PMAxx™-Triton X-100 treatment (Fig. 4a, Table 4). The majority (92.6%, 25/27) of the GII positive final effluent samples had a 100% decline in RNA after the treatment (Fig. 4b). The remaining two samples had an average reduction of 1.86 × 10^4^ ± 2.34 × 10^4^ gc/L. Unlike WWTW1, WWTW2 samples had no increase in measured norovirus concentration after PMAxx™-Triton X-100 treatment.

## Discussion

Randazzo et al. (2016) reported that a combination of PMAxx™ and Triton X-100 improved the reduction of signal from inactivated noroviruses in artificially contaminated lettuce and naturally contaminated irrigation water. Therefore in this study, norovirus GII.4 was used to assess the efficacy of PMAxx™ to distinguish between viable and inactivated viruses when used alone or in combination with Triton X-100. When PMAxx™ was used alone, significant differences in Ct values were observed in both the no-heat-treatment (1.7 log increase) and heat-inactivated groups (1.8 log increase). A higher concentration of PMAxx™ and longer incubation periods may have yielded a larger difference in Ct values between the two sub-groups as previously reported (Codony et al., 2020). Addition of Triton X-100 significantly improved PMAxx™’s performance in the heat-inactivated group (2.7 log increase in Ct value). This suggests that Triton X-100 improves the penetration of damaged virions by PMAxx™, facilitating inhibition of nucleic acid amplification. Mengovirus was not used as a process control but as an extraction control. The detected mengovirus concentrations were only used to determine extraction efficiencies and not to adjust the detected norovirus concentrations as this would distort the data as these viruses have different extraction efficiencies in different sample matrices (Haramoto et al., 2018).

The quantification data showed a significant difference in the mean concentrations of both norovirus GI and GII between raw sewage and final effluent samples from WWTW1. This implies that wastewater treatment methods used at the plant successfully reduced the amount of noroviruses. However, vPCR data showed that a notable amount (34.9%) of the final effluent samples from this site still had RNA from potentially infectious viruses. Interestingly, some of these samples showed an increase in norovirus concentrations after PMAxx™-Triton X-100 treatment. Inter-assay variation could account for this observation. There was no significant difference observed in the reduction rates of RNA between norovirus GI and GII in the final effluent samples. Additionally, no notable difference was detected in the measured concentrations of GI and GII after the PMAxx™-Triton X-100 treatment. At WWTW2, both GI and GII concentrations were lower in final effluent compared to raw sewage and there was no significant difference in average concentration of these viruses in both types of wastewater samples. Norovirus GII was detected in only 7% of samples after vPCR (Supplementary Table S2). This indicates that treatment methods applied by WWTW2 successfully inactivated human noroviruses and therefore their final effluent is less likely to be a risk for norovirus infection. The majority (7/10) of raw sewage samples subjected to vPCR remained positive for norovirus GI, GII or both. This indicates that viruses flowing into the treatment works were intact and potentially infectious prior to the wastewater treatment processes.

Overall, both sampling sites had similar average concentrations of norovirus GI in raw sewage over the study period. However, WWTW1 had a significantly higher average concentration of norovirus GII compared to WWTW2. No significant difference was observed in the average norovirus concentrations in final effluent samples between the sampling sites when RT-qPCR was used. However, after PMAxx™-Triton X-100 treatment, it became evident that WWTW1 had significantly higher concentrations of potentially infectious noroviruses compared to WWTW2. This difference could be due to a number of reasons including but not limited to different population sizes, socioeconomic status, number of infected people between the two populations, amount of run-off water and industrial waste received, and different operating capacities of the WWTWs. However, the most notable factor is the deterioration of WWTW1 due to the on-going operational malfunction due to power cuts, vandalism of infrastructure and limited financial investment to upgrade the facilities as detailed in the 2023 Green Drop Watch Report by the SA Department of Water and Sanitation (DWS, 2023b). These deteriorations can be traced back as far as early 2012 (Zulu, 2020) and are not unique to WWTW1, since WWTW2, which is one of the most compliant plants in the Gauteng province (DWS, 2014) is also negatively impacted (DWS, 2023b).

In conclusion, PMAxx™-Triton X-100 vPCR provides a more accurate reflection of discharge of potentially viable noroviruses in the environment than standard RT-qPCR. The study showed that wastewater treatment methods did reduce the concentration of potentially infectious viruses in final effluent samples. However, due to the high initial load in raw wastewater and low infectious dose of noroviruses, the levels of potentially viable viruses in final effluent is still of concern. The data obtained from this study provided insights into the potential risks posed by poorly treated wastewater and wastewater discharge and adds to the growing body of knowledge on the use of vPCR in assessing the viability of virions detected in final effluents being released into the environment.
